# *In vitro *glucocorticoid sensitivity is associated with clinical glucocorticoid therapy outcome in rheumatoid arthritis

**DOI:** 10.1186/ar4029

**Published:** 2012-08-24

**Authors:** Rogier AM Quax, Jan W Koper, Pascal HP de Jong, Ramona van Heerebeek, Angelique E Weel, Anne M Huisman, Derkjen van Zeben, Frank H de Jong, Steven WJ Lamberts, Johanna MW Hazes, Richard A Feelders

**Affiliations:** 1 Department of Internal Medicine, Erasmus MC, University Medical Center, 's-Gravendijkwal 230, Rotterdam, 3015 CE, The Netherlands; 2 Department of Rheumatology, Erasmus MC, University Medical Center, 's-Gravendijkwal 230, Rotterdam, 3015 CE, The Netherlands; 3 Department of Rheumatology, Maasstad Hospital, Maasstadweg 21, Rotterdam, 3079DZ, The Netherlands; 4 Department of Rheumatology, Sint Franciscus Gasthuis, Kleiweg 500, Rotterdam, 3045PM, The Netherlands

## Abstract

**Introduction:**

Genetic and disease-related factors give rise to a wide spectrum of glucocorticoid (GC) sensitivity in rheumatoid arthritis (RA). In clinical practice, GC treatment is not adapted to these differences in GC sensitivity. *In vitro *assessment of GC sensitivity before the start of therapy could allow more individualized GC therapy. The aim of the study was to investigate the association between *in vitro *and *in vivo *GC sensitivity in RA.

**Methods:**

Thirty-eight early and 37 established RA patients were prospectively studied. *In vitro *GC sensitivity was assessed with dexamethasone-induced effects on interleukin-2 (IL-2) and glucocorticoid-induced leucine zipper (GILZ) messenger RNA expression in peripheral blood mononuclear cells (PBMCs). A whole-cell dexamethasone-binding assay was used to measure number and affinity (1/K_D_) of glucocorticoid receptors (GRs).

*In vivo *GC sensitivity was determined by measuring the disease activity score (DAS) and health assessment questionnaire disability index (HAQ-DI) score before and after 2 weeks of standardized GC treatment.

**Results:**

GR number was positively correlated with improvement in DAS. IL-2-EC_50 _and GILZ-EC_50 _values both had weak near-significant correlations with clinical improvement in DAS in intramuscularly treated patients only. HAQ responders had lower GILZ-EC_50 _values and higher GR number and K_D_.

**Conclusions:**

Baseline cellular *in vitro *glucocorticoid sensitivity is modestly associated with *in vivo *improvement in DAS and HAQ-DI score after GC bridging therapy in RA. Further studies are needed to evaluate whether *in vitro *GC sensitivity may support the development of tailor-made GC therapy in RA.

## Introduction

Rheumatoid arthritis (RA) is a common autoimmune disorder characterized by chronic synovial inflammation, leading to joint destructions. Based on their antiinflammatory properties, glucocorticoids (GCs) have an important role in first-line treatment regimens for RA in combination with disease-modifying antirheumatic drugs (DMARDs). However, on administration of GCs, a wide spectrum of clinical responses is observed with up to 30% of patients being relatively GC resistant [1-3]. In addition, it is well known that in some patients side effects rapidly develop during GC therapy, whereas others tolerate GC well, independent of dose and treatment duration. This indicates that GC sensitivity is highly variable among patients.

Determinants of individual GC sensitivity include both genetic and acquired factors. Functional polymorphisms of the *glucocorticoid receptor (GR) *gene have been identified that modulate GC sensitivity [4]. Recently we found that these polymorphisms are also associated with RA susceptibility and disease severity [5]. Acquired, disease-related factors include the effects of inflammation, mediated by proinflammatory cytokines, on cellular GC sensitivity, resulting in systemic or tissue-specific GC resistance of immunocompetent cells at the site of inflammation [6].

Despite this wide variety in individual GC sensitivity, RA patients are mostly treated with standardized schedules, by using fixed GC dose and treatment duration, inevitably leading to under- or overtreatment in subsets of patients.

Considering the detrimental effects of prolonged synovial inflammation in undertreated patients and the potential severe burden of GC side effects in overtreated patients, it is obvious that a need exists for tools measuring individual GC sensitivity, allowing more tailor-made GC therapy.

GC binding capacity (that is, number and affinity of GRs) has proven its potential as a possible predictor of GC therapy outcome, as has been shown for asthma [7], systemic lupus erythematosus (SLE) [8], and leukemia [9]. In RA, both higher and lower GR expression levels have been reported [10-13]. With respect to *in vivo *GC therapy outcome, Huisman and co-workers [11] showed that GR levels at baseline do not correlate with clinical or radiologic outcome after 2 years of GC therapy. However, this outcome may have been influenced by concomitant use of other antirheumatic drugs.

In addition, studies in patients with inflammatory bowel disease [14], asthma [15], and RA [16] by using *in vitro *functional assays have shown that the degree of GC-mediated suppression of proliferation of peripheral blood mononuclear cells (PBMCs) may predict *in vivo *GC sensitivity. More recently, a diminished inhibitory effect of GCs on PBMC proliferation *in vitro *was shown in a larger cohort of GC resistant RA patients [3].

Recently, we developed *in vitro *bioassays to measure individual cellular GC sensitivity [17]. In these bioassays, dexamethasone-regulated expression of interleukin-2 (IL-2) and glucocorticoid-induced leucine zipper (GILZ) are measured. Transrepressive effects of GC, traditionally considered to be the predominant mechanism regulating antiinflammatory actions of GC, are represented by the IL-2 assay. The GILZ assay is an example of genes in which transcription is transactivated by GCs. Originally such genes were postulated to be responsible for the development of GC-induced side-effects [18,19]. By using these bioassays, a spectrum of GC sensitivity could be demonstrated in healthy individuals.

The aim of this study was to examine whether *in vitro *assessment of GC sensitivity of PBMCs, using both these bioassays and measurement of GC binding capacity, is associated with the *in vivo *response to GC treatment in patients with RA.

## Materials and methods

### Patients

This study was embedded in a multicenter randomized clinical trial studying persons older than 18 years presenting with recent-onset arthritis, the so-called tREACH study (***t***reatment in the ***R***otterdam ***e***arly ***a***rthritis ***c***o***h***ort) [20]. The primary aim of this study is to establish the best treatment strategy for patients with early arthritis.

**Figure 1:**
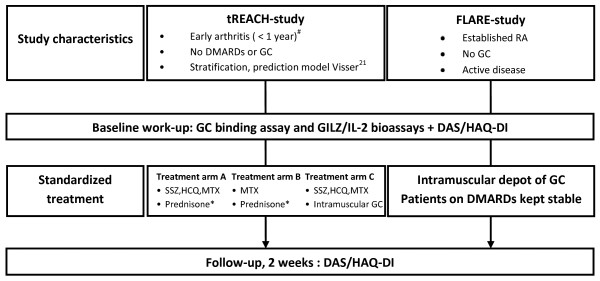
**Flow chart of the tREACH and FLARE study**. The baseline work-up in tREACH patients included the GILZ/IL-2 assays. In FLARE patients, both a GC-binding assay and GILZ/IL-2 assays could be performed. In all patients, disease activity score (DAS) was measured at baseline and after 2 weeks of GC bridging therapy. ^#^Only patients in the high-probability group eventually fulfilling the 1987 ACR criteria for RA were included in the final analysis. *Prednisone is tapered according to the following schedule: weeks 1 through 4, 15 mg/day, weeks 5 and 6, 10 mg/day; week 7 and 8, 5 mg/day; weeks 9 and 10, 2.5 mg/day. Intramuscular GCs could be either methylprednisolone, 120 mg, or triamcinolone acetonide, 80 mg. Baseline workup and start of standardized treatment occurred on the same day. GC, glucocorticoids; HAQ-DI, health assessment questionnaire disability index; HCQ, hydroxychloroquine; MTX, methotrexate; SSZ, sulfasalazine.

Patients were included if arthritis in at least one joint was observed by a rheumatologist, and complaints were present for less than 12 months. With a prediction model developed by Visser *et al. *[21], patients were stratified according to their risk of having persistent erosive disease after a follow-up period of 2 years (high, intermediate, and low probability). We studied 41 patients in the high-probability group. These patients were randomized to three different treatment strategies, all including GC, either oral GC (15 mg prednisone/day, two treatment arms) or intramuscular GC (single depot of methylprednisolone, 120 mg, or triamcinolone acetonide, 80 mg, one treatment arm). All tREACH patients were naïve to GCs and DMARDs (Figure 1).

After a minimum of 1 year of follow-up, the diagnosis of the patients was verified in medical documentation or, if necessary, in consultation with the treating rheumatologist.

In an independent cohort, 37 patients with established RA and active disease (FLARE study) were recruited. Active disease was defined as disease activity requiring GC therapy according to the treating rheumatologists [22]. All patients received a single intramuscular depot of GC (methylprednisolone, 120 mg, or triamcinolone acetonide, 80 mg). None of the FLARE patients had used GC in the last 3 months and were taking stable DMARD therapy (Figure 1). As a control group, we studied healthy laboratory employees (*n *= 20). None of the controls was using a GC.

Of the 41 high-probability patients included via the tREACH study, 38 were ultimately diagnosed as having definite RA. In this group of early RA, two patients were lost to follow-up, leaving 36 patients for complete analysis. After randomization, oral GCs were prescribed to 22 patients, and 14 patients were given a single depot of intramuscular GC. In the FLARE study, two patients were lost to follow-up for logistic reasons. In 10 patients, only one of the assays could be performed due to limited amount of PBMCs. Ultimately, 32 patients could be evaluated for binding capacity of the GC receptor, and 32 patients for the bioassay (in 27 patients, both assays were performed). Patients lost to follow-up were included in the baseline analysis (two patients in each cohort).

## Methods

### Assessment of *in vitro *glucocorticoid sensitivity

In the tREACH cohort, *in vitro *GC sensitivity was assessed with the GC bioassays (for logistic reasons, only enough PBMCs were available for the GC bioassays). In patients participating in the FLARE study, *in vitro *GC sensitivity was assessed by both the GC bioassays and GC binding capacity.

The GC bioassays were performed as described previously [17]. In short, peripheral blood was drawn in all patients before start of treatment by using Cell Preparation Tubes with Sodium Heparin (Becton Dickinson, Breda, The Netherlands), allowing isolation of PBMCs. Cells were resuspended in RPMI 1640 medium containing L-glutamine supplemented with penicillin (100 U/ml) and streptomycin (100 μg/ml) and 10% fetal calf serum (FCS) and precultured overnight in a 48-well plate (Costar, Amsterdam, The Netherlands, 5.0 × 10^5 ^cells/well in duplicate, density of 4.0 × 10^6^/ml). A single batch of FCS was used throughout. Before use, this batch was analyzed for cortisol content, which was found to be below the detection limits.

Trypan blue staining revealed the viability of isolated cells to be greater than 95%. The next day, cells were incubated with dexamethasone 0, 0.33, 1, 3.3, 10, 33, 100, and 333 n*M *dexamethasone and stimulated with 10 μg/ml phytohemagglutinin (Sigma-Aldrich, Zwijndrecht, The Netherlands). After 4 hours in the incubator, total RNA of the cells was collected (Total RNA isolation Kit, Roche, Almere, The Netherlands). Reverse transcription was performed by using 100 ng total RNA per reaction. Quantitative real-time PCR analysis was carried out on a 7900HT Taqman machine (Applied Biosystems, Nieuwerkerk aan den IJssel, The Netherlands), according to the manufacturer's instructions. Data were analyzed by using the SDS 2.4 software (Applied Biosystems). GC-specific transactivation of the *GILZ *gene and transrepression of the *IL-2 *gene were measured while correcting for the housekeeping gene hypoxanthine phosphoribosyltransferase (HPRT) by using the ΔΔCT method; primers and probes were obtained from Biolegio, Nijmegen, The Netherlands (see Additional File 1 Table S1). Half-maximal effective concentration (EC_50_) was calculated by using nonlinear regression in GraphPad Prism 5.0 and used as a read-out for *in vitro *GC sensitivity. The EC_50 _values of GILZ and IL-2 in PBMCs were not significantly influenced by the cellular composition (percentages lymphocytes and monocytes) of the PBMCs (data not shown).

GC binding capacity was measured by using a whole-cell dexamethasone-binding assay, as described previously, with minor modifications [23]. In brief, by using PBMCs from the same isolation procedure, incubation was started in a volume of 200 μl (0.5 to 2 × 10^6 ^cells) containing [^3^H] dexamethasone at concentrations of 1 to 30 n*M *with and without a 400-fold excess of unlabeled dexamethasone reflecting nonspecific and total binding of [^3^H] dexamethasone, respectively. Two tubes without labeled dexamethasone were incubated under the same conditions for determination of cell number and viability at the end of the procedure. The PBMCs were incubated during 1 hour at 30°C in a shaking water bath. The incubation was stopped by the addition of 2 ml cold saline, followed by centrifugation and two washing steps. Finally, the PBMCs were resuspended in 250 μl saline. Radioactivity in 200 μl of this suspension was counted in a liquid scintillation counter. Specific binding was calculated by subtracting nonspecific binding from total binding. EC_50 _values, receptor number, and ligand affinity (1/K_D_) were calculated by using the nonlinear regression method (GraphPad Prism, version 5.0; La Jolla, CA, USA).

### *In vivo *glucocorticoid sensitivity

Trained research nurses examined patients before and after 2 weeks of standardized GC treatment. Disease Activity Score (DAS, 44 joints) was calculated according to the following formula: DAS = 0.54 × √RAI + 0.065 × SJC44 + 0.33 × ln(ESR) + 0.007 × GH (RAI, Ritchie Articular Index; SJC44, 44 swollen-joint count; ESR, erythrocyte sedimentation rate; GH, general health on a 100-mm scale). As primary outcome, the relative decrease in DAS (100 × ((DAS_baseline _- DAS_after 2 weeks_)/DAS_baseline_)) was used as an index for *in vivo *GC sensitivity. By using this continuous outcome variable, a floor effect in patients with relatively low disease activity was prevented. In addition, continuous variables represent the full information, in contrast to (arbitrary) categoric data (that is, response criteria). We chose a 2-week interval for follow-up in tREACH patients to minimize the influence of the disease-modifying effects of the other antirheumatic drugs on the DAS. A similar follow-up period was chosen in the FLARE study to make comparisons between the groups possible. During the study period, the dose of DMARD(s) already being used was not changed, and no additional anti-rheumatic therapy was started.

To explore further the effectiveness of GC therapy, the impact of GC treatment on performing activities of daily living was assessed by using the health assessment questionnaire disability index score (HAQ-DI). The HAQ-DI is a widely used and validated tool to quantify functional disability in RA [24] and comprises questions about different aspects of daily life. In particular, the minimal import difference (MID) in the HAQ-DI score is the smallest difference in HAQ-DI score that patients sense as a difference. In clinical trials, the MID in HAQ-DI improvement ranged from 0.22 to 0.24 [16,17]. As a result, patients were classified as responder (HAQ-DI_baseline _- HAQ-DI_2wks _≥ 0.25) or nonresponder (HAQ-DI_baseline _- HAQ-DI_2wks _< 0.25).

### Glucocorticoid-induced side effects

We measured blood pressure and body weight before and after 3 months of GC therapy in tREACH patients. Furthermore, glycosylated hemoglobin (HbA_1c_) was measured at baseline and after 3 months in tREACH patients (HbA_1c _analyzer, type Adams A1c HA-8160, Menarini Benelux).

### Statistical analysis

Differences in continuous variables between the cohorts were tested by using analysis of variance (ANOVA). GILZ-EC_50 _values were normally distributed (Kolmogorov-Smirnoff *P *> 0.20), whereas IL-2-EC_50 _was square-root transformed, and the number of receptors and K_D _were both natural logarithm transformed to normalize the data. Bonferroni *post hoc *tests were used to correct for multiple testing.

Pearson or Spearman correlation coefficients were used to describe the bivariate relations between *in vitro *parameters of GC sensitivity and DAS at baseline and relative decrease in DAS.

ANOVA analysis was applied to test for differences in *in vitro *parameters of GC sensitivity between HAQ responders and nonresponders. Paired *t *tests or Wilcoxon Signed Ranks Tests were used for analysis of alterations in DAS, HAQ-DI scores, blood pressure, body weight, and HbA_1c _values.

To test for potential confounders, each of the individual *in vitro *parameters of GC sensitivity (that is, IL-2- and GILZ-EC_50_, K_D_, and number of GRs) and selected covariates were modeled by using linear regression (relative decrease in DAS as the dependent variable). These selected covariates included gender, age, and, based on potential synergistic immunomodulating properties with GCs, use of NSAIDs, number of DMARDs, and use of anti-TNF-α agents.

Orally and intramuscularly treated patients were analyzed separately because of nonequivalent cumulative dosages of GC (cumulative GC dosage: oral > intramuscular). We considered differences statistically significant if *P *≤ 0.05 (two-sided).

### Ethical approval

All subjects signed informed consent, and the study was approved by the medical ethics committee of the Erasmus Medical Center.

## Results

Thirty-eight tREACH patients and 37 FLARE patients were prospectively studied. Patients in the FLARE study had a significantly higher disease activity at baseline, a longer duration of disease, and a higher percentage of erosions compared with tREACH patients. Further baseline characteristics are summarized in Table 1.

**Table 1:** Patient characteristics

	Controls (*n *= 20)	TREACH (*n *= 38)	FLARE (*n *= 37)
Female gender, *n *(%)	10 (50)	25 (65.8)	25 (67.6)

Age in years, mean (SD)	31.8 (9.7)	53.3 (13.98)^a^	53.7 (13.40)^a^

Disease duration in months, median (range)	**-**	5.4 (2-12)	73.0 (0-414)^b^

Presence of joint erosions, *n *(%)	**-**	10 (26.3)	20 (54.1)^c^

Anti-CCP positive, *n *(%)	**-**	30 (78.9)	24 (85.7)^d^

Rheumatoid factor (IgM) positive, *n *(%)	**-**	31 (81.6)	27 (73.0)

DAS44 at baseline, mean (SD)	**-**	3.05 (0.92)	3.57 (0.95)^c^

HAQ-DI at baseline, mean (SD)	-	-	1.43 (0.62)

Use of NSAID, *n *(%)	-	25 (67.6)	19 (51.4)

Use of methotrexate, *n *(%)	-	-	22 (59.5)

Use of hydroxychloroquine, *n *(%)	-	-	11 (29.7)

Use of sulfasalazine, *n *(%)	-	-	5 (13.5)

Number of DMARDs, median (range)	-	-	1 (0-3)^e^

Use of anti-TNF-α therapy, *n *(%)	-	-	5 (13.5)

DAS44, Disease Activity Score, 44 joints; HAQ-DI, Health Assessment Questionnaire Disability Index. ^a^*P *< 0.001 as compared with healthy controls; ^b^*P *< 0.001 as compared with tREACH patients; ^c^*P *< 0.05; ^d^anti-CCP was not routinely analyzed; percentage is based on 28 patients with known anti-CCP status; ^e^Seven patients were not using any DMARD at the time of assessment.

### Baseline *in vitro *glucocorticoid sensitivity in RA and healthy controls

**Figure 2:**
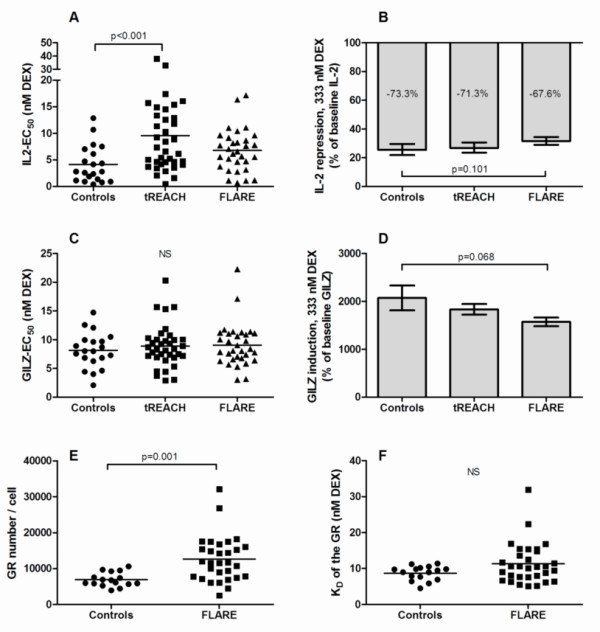
**Baseline *in vitro *parameters of GC sensitivity in healthy controls, tREACH, and FLARE patients**. The IL-2 assay **(A) **and GILZ-assay **(C) **were performed in both tREACH and FLARE patients (bioassays in 32 patients, GC-binding assay in 32 patients, bioassays and GC-binding assay in 27 patients; control groups for the bioassays (*n *= 20) and binding assay (*n *= 16) were not the same). As secondary outcome, IL-2 repression **(B) **and GILZ induction **(D) **was calculated as follows: $IL-2\;repression=100\times \frac{\text{(IL}2-\text{expression},\;\text{PHA)}-\text{(IL2}-\text{expression,}\;\text{333nM)}}{\left(IL2-expression,\;PHA\right)}$$GILZ\;induction=100\times \frac{\text{(GILZ}-\text{expression, 333nM)}}{\text{(GILZ}-\text{expression, PHA)}}$ The numbers of GRs **(E) **and the affinity of the receptor **(F) **were determined in FLARE patients only. EC_50_, half maximal effective concentration. *P *values were calculated by using ANOVA and Bonferroni *post hoc *correction; normalized data were used where appropriate.

Overall, patients with early (tREACH cohort) and established RA (FLARE cohort) had higher mean EC_50 _values in the IL-2 assay than did healthy controls (although not statistically significant in the FLARE cohort), indicating that RA patients needed a higher dosage of dexamethasone to suppress IL-2 mRNA expression *in vitro*. In contrast to this, similar EC_50 _values were measured in the GILZ assay (Figure 2A, C). Patients participating in the FLARE study had a higher number of GRs compared with healthy controls, while having comparable affinity (1/K_D_) of the receptor (Figure 2E, F). The percentage of monocytes was measured in subsets of FLARE patients and healthy controls and did not differ significantly (mean ± SD, 24.6 ± 9.2 in FLARE patients versus 20.9 ± 5.0 in healthy controls). Ligand affinity of monocytes and lymphocytes did not differ significantly. The number of glucocorticoid receptors per cell was about threefold higher in monocytes as compared with lymphocytes (data not shown). The maximum induction of GILZ and repression of IL-2 tended to be lower in the established RA cohort as compared with healthy controls (*P *= 0.068 and *P *= 0.101, respectively) (Figure 2B, D).

No correlations were found between the DAS and parameters of *in vitro *GC sensitivity. Of the variables used to calculate the DAS, a negative association was observed between the RAI and IL-2-EC_50 _(*ρ *= -0.465; *P *= 0.005), but only in the early RA patients. No gender differences were noted at the mean level of the IL-2-EC_50 _and GILZ-EC_50_, number of GRs, or the affinity of the receptor.

HAQ-DI sum scores before start of treatment did not show any correlations with *in vitro *parameters of GC sensitivity. Male and female individuals did not differ significantly in HAQ-DI sum scores.

### Correlation between *in vitro *parameters of glucocorticoid sensitivity

GILZ-EC_50 _and IL-2-EC_50 _were positively correlated, but only in the patients with early RA (*ρ *= 0.383; *P *= 0.028). In patients with established RA, the number of GRs was inversely correlated with GILZ-EC_50 _and IL-2-EC_50 _(*ρ *= -0.401; *P *= 0.042; and *ρ *= -0.462; *P *= 0.020 respectively). K_D _was also inversely correlated with GILZ-EC_50 _(*ρ *= -0.413; *P *= 0.032), but not with IL-2-EC_50_. Finally, K_D _and GR-number were correlated (*ρ *= 0.627; *P *< 0.001; see Additional File 2 Figure S2).

### Pretreatment *in vitro *glucocorticoid sensitivity and disease activity in RA after 2 weeks of glucocorticoid therapy

After 2 weeks of GC treatment, a significant decrease in disease activity was measured in both orally and intramuscularly treated patients (ΔDAS_oral _= 0.92; *P *< 0.001; ΔDAS_intramuscular _= 0.89; *P *< 0.001; and see Additional File 3 Table S3). The interquartile range in relative decrease in DAS was 22% and 43% in orally and intramuscularly treated patients, respectively, indicating greater variability in relative decrease in DAS in the intramuscularly treated group.

**Figure 3:**
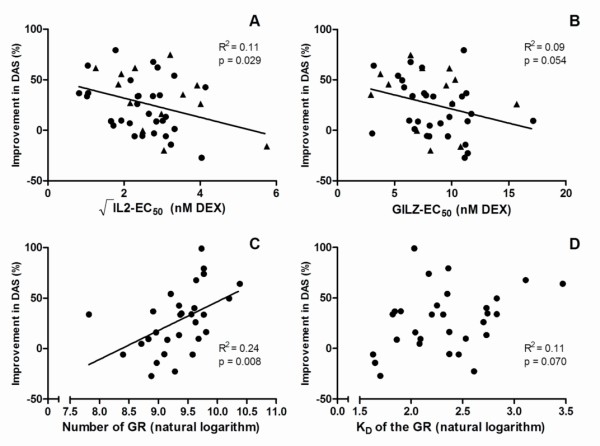
**Correlation between *in vitro *and *in vivo *glucocorticoid sensitivity in intramuscularly treated RA patients**. *In vivo *glucocorticoid sensitivity is presented as percentage improvement in DAS according to the following formula: $100\times \frac{DAS,baseline-DAS,after\;2\;weeks}{DAS,baseline}$ Correlations between √IL-2-EC_50 _values **(A)**, GILZ-EC_50 _values **(B)**, natural logarithm of the number of GRs per cell **(C)**, and natural logarithm of the K_D _of the receptor **(D) **and percentage improvement DAS. *R*^2^, square of the Pearson correlation coefficient; proportion explained variability. Triangles (▲) represent the tREACH patients, and solid circles (●) represent FLARE patients.

In patients treated with a single intramuscular depot of GC (all FLARE patients and a proportion of tREACH patients), a modest inverse relation was found between *in vitro *GC sensitivity as reflected by IL-2-EC_50 _values and the percentage improvement in DAS after 2 weeks (*P *= 0.029; Figure 3A). Similarly, near-significance was reached for GILZ-EC_50 _values and the relative decrease in DAS, but also only in intramuscularly treated patients (*P *= 0.054; Figure 3B). In addition, the number of GRs displayed a modest positive relation with the improvement in DAS in patients with intramuscular depots of GC, and a positive trend was observed for the K_D _of the GRs (*P *= 0.008 and *P *= 0.070, respectively; Figure 3C, D).

With multiple regression, however, both the number of GRs and K_D _of the receptor were significant factors contributing to the relative decrease in DAS. The negative association between IL-2-EC_50 _and GILZ-EC_50 _values and relative decrease in DAS persisted, although only near-significance was reached (Table 2).

**Table 2:** *In vitro *parameters of GC sensitivity and relative decrease in DAS

**Bioassays**			**GC binding assay**		
	
	**β (95% CI)^a^**	***P *value**		**β (95% CI)^a^**	***P *value**
	
IL-2-EC_50_	-0.014 (-0.028-0.001)	0.058	K_D_	0.03 (0.014-0.046)	0.001
	
Age	0.005 (-0.002-0.110)	0.161	Age	0.009 (0.002-0.017)	0.020
Gender	0.128 (-0.057-0.312)	0.169	Gender	0.199 (0.013-0.385)	0.037
Use of NSAID	0.087 (-0.087-0.261)	0.316	Use of NSAID	0.212 (0.025-0.400)	0.028
Number of DMARDs	0.023 (-0.079-0.125)	0.647	Number of DMARDs	0.112 (0.004-0.221)	0.043
Use of anti-TNF-α	0.008 (-0.283-0.299)	0.955	Use of anti-TNF-α	0.222 (-0.010-0.455)	0.060
	
GILZ-EC_50_	-0.023 (-0.046-0.001)	0.062	GR number/1,000	0.027 (0.012-0.042)	0.001
	
Age	0.006 (-0.001-0.014)	0.079	Age	0.010 (0.002-0.018)	0.015
Gender	0.116 (-0.075-0.308)	0.225	Gender	0.017 (-0.188-0.223)	0.862
Use of NSAID	0.181 (-0.004-0.366)	0.055	Use of NSAID	0.203 (0.006-0.400)	0.044
Number of DMARDs	0.021 (-0.085-0.126)	0.695	Number of DMARDs	0.084 (-0.027-0.195)	0.132
Use of anti-TNF-α	0.023 (-0.270-0.320)	0.874	Use of anti-TNF-α	0.129 (-0.121-0.378)	0.297

In all four models, relative decrease in DAS was the dependent variable. The data represent the combined bioassays from intramuscularly treated patients with early (*n *= 14) and established RA (*n *= 31). GILZ-EC_50 _and IL-2-EC_50 _were not associated with relative decrease in DAS in orally treated patients in recent-onset RA (*n *= 22). The GC-binding assay is performed only in established RA (*n *= 30, all intramuscular GC). **^a^**Values represent adjusted β-coefficients and 95% confidence intervals.

Of note, in the subgroup of patients with evaluation of GC-binding capacity (FLARE study), age and use of NSAIDs were also independent predictors of improvement of disease activity after 2 weeks of GC treatment. Age and use of NSAIDs both had positive β coefficients, indicating a better response with older age and use of NSAIDs.

### Pretreatment *in vitro *glucocorticoid sensitivity and functional disability in RA after 2 weeks of glucocorticoid therapy

**Figure 4:**
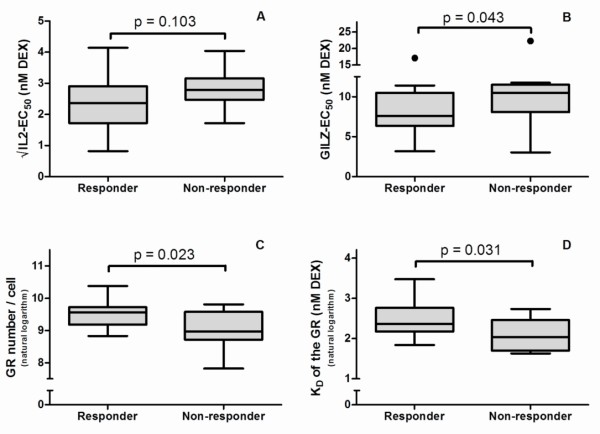
***In vitro *glucocorticoid sensitivity and improvement in HAQ-DI score in patients with established rheumatoid arthritis (FLARE study)**. Boxplots (each box shows the mean and interquartiles) and outliers (●) of IL-2-EC_50 _**(A)**, GILZ-EC_50 _**(B)**, number of GRs **(C) **and K_D _of the GRs **(D) **in HAQ-DI responders and HAQ-DI-nonresponders. Patients are defined as responders if their HAQ-DI sum scores were at least 0.25 lower after GC therapy.

After 2 weeks of GC treatment, a significant decrease in HAQ-DI sum scores was measured (ΔHAQ-DI = -0.40; *P *< 0.001). However, 12 of 34 patients still had to be classified as nonresponders. Responders had lower EC_50 _values of GILZ and higher numbers of GRs with higher K_D _(Figure 4). IL-2-EC_50 _values tended to be lower in responders.

### *In vitro *glucocorticoid sensitivity and development of glucocorticoid-mediated metabolic side effects

The mean systolic and diastolic blood pressure of patients was reduced at their 3-month follow-up visit (systolic RR_baseline_, 145.0 mm Hg; systolic RR_3 months_, 134.4 mm Hg; *P *= 0.005; and diastolic RR_baseline_, 87.4 mm Hg; diastolic RR_3 months_, 82.6 mm Hg; *P *= 0.003). This decrease was observed in both orally and intramuscularly treated patients. Body mass index did not change significantly after 3 months (BMI_baseline_, 26.9; BMI_3 months_, 26.7).

At baseline, the mean HbA_1C _was 5.51% (reference range, 4.5% to 6.0%). Three patients had HbA_1C _values above the upper limit of the normal range. After 3 months, the mean HbA_1C _was even somewhat lower (5.31%; *P *= 0.016). Nine patients had a higher HbA_1C_, four patients had an equal percentage of HbA_1C_, and 13 patients had an improvement. No relation was found between alterations in HbA_1C _and blood pressure and *in vitro *GC sensitivity, as measured by the bioassay.

## Discussion

We examined whether *in vitro *GC sensitivity is associated with the clinical response to GC treatment in RA. Our results show that, in particular, the number of GRs in PBMCs and the K_D _of the GRs correlated with *in vivo *GC sensitivity, as reflected by the relative decrease in DAS. Near-significant associations were found between dexamethasone-mediated changes in IL-2- and GILZ-mRNA expression levels and the relative decrease in DAS. Similar patterns between clinically relevant improvement in HAQ-DI sum scores and *in vitro *parameters of GC sensitivity were observed.

Remarkably, PBMCs of RA patients have a decreased *in vitro *capacity for transrepression, which is most pronounced in the early RA cohort. This transrepression of proinflammatory cytokine production by (endogenous) GC is an important mechanism to counteract the inflammatory response [25]. Consequently, reduced transrepression might hamper the resolution of acute inflammation, governing the evolution into a chronic phase of inflammation, a central feature of many autoimmune diseases. Interestingly, polymorphisms of the GR gene associated with reduced (that is, 9β) or increased (that is, Bcll and N363S) GC sensitivity are associated with increased respectively decreased susceptibility to RA [5]. Next to decreased GC sensitivity, a blunted hypothalamic-pituitary-adrenal axis has been postulated to be part of the pathophysiology of RA [26].

Importantly, we did not find a relation between disease activity and *in vitro *GC sensitivity, suggesting that the impaired GC sensitivity is not just due to increased levels of proinflammatory cytokines. This is in accordance with the study performed by Hearing and co-workers [14], who also did not find a relation between disease activity and *in vitro *GC sensitivity in inflammatory bowel disease.

In contrast to this reduced GC sensitivity at the transcriptional level, we found a higher number of GRs in patients with established RA. A large study by Schlaghecke *et al. *[13] showed lower numbers of GR in RA. In contrast, Eggert and co-workers [10] found increased expression of GR, which dramatically decreased after long-term GC treatment. Interestingly, the only study with longitudinal data on GR expression in RA reports an increase in GR expression over time in female RA patients, suggesting a compensatory mechanism for the ongoing inflammatory state [11]. In addition to this concept, the higher numbers of GRs in our cohort might be interpreted as a counterbalancing mechanism for the reduced GC sensitivity. In line with this hypothesis, we found a correlation between higher numbers of GRs and lower EC_50 _values of GILZ and IL-2.

GC exert their antiinflammatory properties via the GR. On binding of GC to the GRs, the receptor-ligand complex migrates to the nucleus to interact with GC-responsive elements of target genes. During inflammation, cellular GC sensitivity can be modulated by cytokines via effects on GR number and affinity, GR translocation to the nucleus, interaction with inflammatory transcription factors (for example, NF-κB, AP-1) and expression of the GR-β splice variant [27]. The assessment of GC-mediated gene expression, as performed in our bioassay, may have the advantage of integrating all postreceptor downstream factors that modulate GC sensitivity. Originally, the immunosuppressive effects of GC were attributed to transrepression of immune genes. We indeed found that IL-2 EC_50 _values are moderately associated with the relative decrease in DAS. However, in the last decade, increasing evidence has been obtained pointing toward immunomodulating effects of GC-activated genes [28].

In this perspective, the GILZ gene studied in our bioassay is of particular interest. GILZ can directly interfere with the AP-1 complex [29] and can also inhibit NF-κB nuclear translocation and DNA binding *in vitro *[30]. Recently, GILZ has been demonstrated to function as an endogenous inhibitor of chronic inflammation in a murine model of RA [31]. In addition, GILZ transgenic mice are less prone to develop T-helper 1-mediated colitis [32]. We extend these observations by demonstrating that GILZ regulation by dexamethasone *in vitro *might be a potential marker for *in vivo *effects of GC therapy in humans.

Remarkably, the predictive value of the GILZ and IL-2 assays is found only in the intramuscularly treated patients and not in the orally treated patients. A possible explanation is that the higher dosage of GCs used in the orally treated patients masks subtle differences in GC sensitivity. This is supported by the fact that the interquartile range in relative decrease in DAS was higher in the intramuscularly treated patients. Furthermore, a lack of compliance in orally treated patients could play a role, whereas this problem is obviously not present in intramuscularly treated patients. Finally, differences in pharmacokinetics and duration of disease could also be causes adding to observed differences between orally and intramuscularly treated patients.

In our group of patients with established RA, both the number of GRs and the K_D _were positively correlated with improvement in disease activity. From a biologic point of view, higher numbers of receptors correlating to better response seems plausible. Indeed, GR levels have been shown to serve as possible markers of GC-therapy outcome in SLE and leukemia [8,9]. Conversely, our observations concerning the K_D _of the GRs are in contrast to other reports [7,33]. In this perspective, it is important to note that higher numbers of GRs were accompanied by lower affinity of the receptor (that is, a higher K_D_) in several other conditions [7,33-37].

Whether this phenomenon truly occurs *in vivo *or represents an artificial correlation (because K_D _and GR number are calculated from the same data) is yet unclear. Analysis of GR number and K_D _separately by using different techniques could possibly give more insight into this intriguing observation. Clearly, the interpretation of binding assays should be done with caution.

Although we did not measure serum levels of the exogenously administered GCs in our patients, the (average) serum concentrations of these GCs, in the doses administrated, are reported to be in the same (equipotent) range as the GILZ and IL-2 EC_50 _values and the K_D _of the GRs, suggesting that *in vitro *parameters of GC sensitivity may reflect *in vivo *GC sensitivity reasonably well [38,39].

Unexpectedly, the IL-2/GILZ assay, integrating all determinants of GC sensitivity up to the transcriptional level, showed a weaker correlation with the *in vivo *response than the more-upstream GR. However, GCs also have effects that do not require gene transcription, also referred to as nongenomic effects of GCs [40]. Also in RA, nongenomic actions are important, as illustrated by rapid inhibition of leukocyte recruitment in inflamed joints after GC administration [41]. GR levels may therefore be a better predictor of *in vivo *GC effects, because both genomic and nongenomic actions of GCs are taken into account.

Our study clearly highlights the potential of *in vitro *(bio) assays as possible clinical markers for GC treatment of RA patients. Recently it was shown that assessment of early arthritis patients by a rheumatologist within 12 weeks was associated with less joint destruction and a higher chance of DMARD-free remission, as compared with patients assessed after this so-called window of opportunity [42]. This favorable outcome of early treatment could be further substantiated by effective (tailor-made) GC treatment in the window of opportunity and emphasizes the need for biomarkers of GC sensitivity before the start of GC treatment.

However, several limitations in our study must be addressed. A relatively weak correlation was found between the GILZ and IL-2 assays and *in vivo *glucocorticoid sensitivity, restricting the usefulness of these assays in the clinical context at this moment. Further, presumably because of the restricted period of GC treatment, we could not evaluate the potency of our bioassay and binding assay to predict susceptibility for GC-mediated side effects. Also, because GC sensitivity is highly tissue specific, extrapolation of our findings to other inflammatory disorders should be done with caution. As prednisone is a pro-drug requiring reduction by 11β-HSD type 1, and methylprednisolone and triamcinolone acetonide are active 11-hydroxysteroids, it is possible that differences in the cortisol-cortisone shuttle, mediated by the proinflammatory state, might also have influenced *in vivo *GC sensitivity [43]. Furthermore local steroid metabolism in the synovial cells may play a role in increasing local cortisol and prednisolone concentration, as shown by Hardy and co-workers [44]. Considering this tissue specificity and the sample size of our RA cohort, validation of these *in vitro *assays should be done in cohorts with both RA and other autoimmune disorders.

## Conclusions

We show that after 2 weeks of GC treatment of patients with RA, the relative decrease in DAS *in vivo *is modestly associated with the number and affinity of GRs. Near-significant associations were found with EC_50 _values of IL-2 and GILZ. *In vitro *identification of hypo- or hypersensitive subgroups of RA patients may facilitate a more (alternatively: may facilitate individual GC therapy) individual GC therapy for these particular patients to maximize therapeutic efficacy and minimize time- and dose-dependent side effects. Further studies evaluating the number and affinity of GRs in PBMCs at baseline in relation to improvement in DAS are needed to establish whether assessment of *in vitro *GC sensitivity can support individualized therapeutic management of RA patients treated with GCs.

## Abbreviations

ANOVA: analysis of variance; DAS: disease activity score; DMARDs: disease-modifying antirheumatic drugs; EC_50_: half-maximal effective concentration; ESR: erythrocyte sedimentation rate; GC: glucocorticoid; GH: general health at a 100-mm scale; GILZ: glucocorticoid-induced leucine zipper; GR: glucocorticoid receptor; HAQ-DI: health assessment questionnaire disability index; HbA_1c_: glycosylated hemoglobin; IL-2: interleukin-2; PBMC: peripheral blood mononuclear cell; RA: rheumatoid arthritis; RAI: Ritchie Articular Index; SJC44: 44 swollen-joint count; tREACH: treatment in the Rotterdam early arthritis cohort.

## Competing interests

The authors declare that they have no competing interests.

## Authors' contributions

RAMQ participated in the study design and carried out the laboratory work, the statistical analysis, and wrote the paper. JWK participated in the study design, laboratory work, co-writing the paper, and research supervision. PHPdeJ, AEW, AMH, and DvZ participated in the study design and collection of patient data. RvH participated in the laboratory work. FHdeJ, SWJL, and JMWH participated in co-writing the paper and research supervision. RAF participated in the study design, co-writing the paper, and research supervision. All authors read and approved the final manuscript.

## Supplementary Material

Additional file 1 **Table S1. Primer and probe sequences for GILZ, IL-2, and HPRT**. This table gives the sequences of the primers and probes used in the bioassay to measure messenger RNA levels of GILZ, IL-2, and HPRT. Click here for file

Additional file 2 **Figure S2. Bivariate correlations between *in vitro *parameters of glucocorticoid sensitivity**. This figure displays how different *in vitro *parameters of GC sensitivity, as measured in the bioassay and GC-binding assay, correlate to each other. Click here for file

Additional file 3 **Table S3. DAS and individual measures of the DAS in tREACH and FLARE patients**. This table provides detailed information on DAS and individual measures of the DAS in the different subsets of studied patients, both at baseline and after 2 weeks of GC treatment. Click here for file
