# Supplementary material for: In vitro glucocorticoid sensitivity is associated with clinical glucocorticoid therapy outcome in rheumatoid arthritis
Source: Arthritis Res Ther. 2012 Aug 24;14(4):R195. doi: 10.1186/ar4029 (PMC3580593; doi:10.1186/ar4029)
Supplement: Additional file 1 — Table S1. Primer and probe sequences for GILZ, IL-2, and HPRT. This table gives the sequences of the primers and probes used in the bioassay to measure messenger RNA levels of GILZ, IL-2, and HPRT. [file ar4029-S1.DOCX]

| Supplementary Table 1. Primer and probe sequences for GILZ, IL-2 and HPRT | | |
| --- | --- | --- |
| GILZ: forward primer |  | 5’-GCACAATTTCTCCATCTCCTTCTT-3’ |
| GILZ: reverse primer |  | 5’- TCAGATGATTCTTCACCAGATCCA -3’ |
| GILZ: probe |  | 5’-6FAM-TCGATCTTGTTGTCTATGGCCACCACG-BHQ1-3’ |
|  |  |  |
| IL-2: forward primer |  | 5’-TTTGAATGGAATTAATAATTACAAGAATCC-3’ |
| IL-2: reverse primer |  | 5’-TCTAGACACTGAAGATGTTTCAGTTCTGT-3’ |
| IL-2: probe |  | 5’-6FAM-CCAGGATGCTCACATTTAAGTTTTACATGCCC-BHQ1-3’ |
|  |  |  |
| HPRT: forward primer |  | 5’-CACTGGCAAAACAATGCAGACT-3’ |
| HPRT: reverse primer |  | 5’-GTCTGGCTTATATCCAACACTTCG T-3’ |
| HPRT: probe |  | 5’-6FAM-CAAGCTTGCGACCTTGACCATCTTTGGA-TAMRA-3’ |
