# Supplementary material for: In vitro glucocorticoid sensitivity is associated with clinical glucocorticoid therapy outcome in rheumatoid arthritis
Source: Arthritis Res Ther. 2012 Aug 24;14(4):R195. doi: 10.1186/ar4029 (PMC3580593; doi:10.1186/ar4029)
Supplement: Additional file 2 — Figure S2. Bivariate correlations between in vitro parameters of glucocorticoid sensitivity. This figure displays how different in vitro parameters of GC sensitivity, as measured in the bioassay and GC-binding assay, correlate to each other. [file ar4029-S2.DOC]

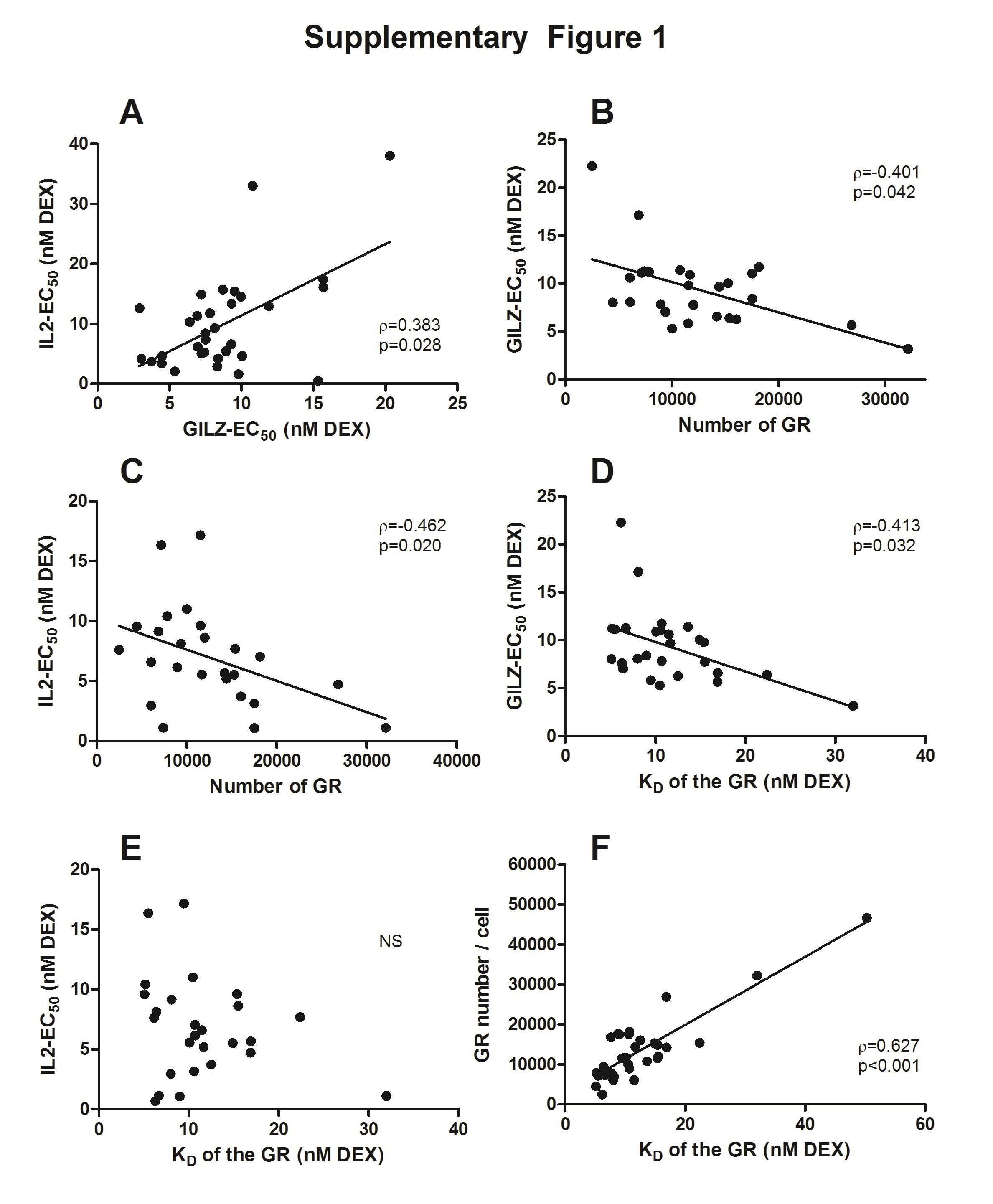


The correlations between IL-2-EC_50_ and GILZ-EC_50_ in early RA (A), GILZ-EC_50_ and number of GR (B), IL-2-EC_50_ and number of GR (C), GILZ-EC_50_ and K_D_ of the GR (D), IL-2-EC_50_ and K_D_ of the GR (E) and K_D_ of the GR and number of GR (F) are depicted.
