# Supplementary material for: In vitro glucocorticoid sensitivity is associated with clinical glucocorticoid therapy outcome in rheumatoid arthritis
Source: Arthritis Res Ther. 2012 Aug 24;14(4):R195. doi: 10.1186/ar4029 (PMC3580593; doi:10.1186/ar4029)
Supplement: Additional file 3 — Table S3. DAS and individual measures of the DAS in tREACH and FLARE patients. This table provides detailed information on DAS and individual measures of the DAS in the different subsets of studied patients, both at baseline and after 2 weeks of GC treatment. [file ar4029-S3.DOCX]

| Supplementary Table 2. DAS and individual measures of the DAS in tREACH and FLARE patients | | | | | | | | |
| --- | --- | --- | --- | --- | --- | --- | --- | --- |
|  | **tREACH** | **p-value** |  | **tREACH** | **p-value** |  | **FLARE** | **p-value** |
|  | *oral GC (N=15)* | *(0-2 wks change)* |  | *intramuscular GC (N=23)* | *(0-2 wks change)* |  | *intramuscular GC (N=37)* | *(0-2 wks change)* |
| DAS, baseline (mean; SD) | 3.12 (1.05) | <0.001 |  | 2.94 (0.69) | <0.001 |  | 3.57 (0.95) | <0.001 |
| DAS, 2 weeks (mean; SD) | 2.20 (0.96) |  |  | 1.84 (0.80) |  |  | 2.70 (1.39) |  |
|  |  |  |  |  |  |  |  |  |
| SJC, baseline | 5 (1-18) | 0.013 |  | 6 (1-19) | 0.013 |  | 7 (2-25) | <0.001 |
| SJC, 2 weeks | 3 (0-11) |  |  | 2 (0-9) |  |  | 3 (0-26) |  |
|  |  |  |  |  |  |  |  |  |
| RAI, baseline | 6 (0-50) | 0.001 |  | 4 (0-9) | 0.048 |  | 7 (0-31) | 0.040 |
| RAI, 2 weeks | 2 (0-19) |  |  | 0 (0-9) |  |  | 6 (0-35) |  |
|  |  |  |  |  |  |  |  |  |
| ESR, baseline | 22 (4-80) | <0.001 |  | 23 (9-69) | 0.142 |  | 22.5 (1-85) | 0.002 |
| ESR, 2 weeks | 13 (1-60) |  |  | 16 (4-69) |  |  | 18 (1-75) |  |
|  |  |  |  |  |  |  |  |  |
| GH, baseline | 53 (9-92) | 0.002 |  | 40 (11-77) | 0.451 |  | 69 (9-99) | <0.001 |
| GH, 2 weeks | 28 (0-70) |  |  | 31 (0-80) |  |  | 50 (3-100) |  |

One patient each in the orally and intramuscularly treated tREACH group was lost-to-follow-up and two patients in the FLARE study did not have a second DAS. Values are given as median (range) unless otherwise stated. SJC: Swollen joint count; RAI: Ritchie Articular Index; ESR: Erytrocyte Sedimentation Rate; GH = general health at a 100 mm scale.
